# Study protocol of the iMPaCT project: a longitudinal cohort study assessing psychological determinants, sexual behaviour and chlamydia (re)infections in heterosexual STI clinic visitors

**DOI:** 10.1186/s12879-018-3498-6

**Published:** 2018-11-13

**Authors:** Daphne A. van Wees, Janneke C. M. Heijne, Titia Heijman, Karlijn C. J. G. Kampman, Karin Westra, Anne de Vries, Mirjam E. E. Kretzschmar, Chantal den Daas

**Affiliations:** 1 Centre for Infectious Disease Control, National Institute for Public Health and the Environment, Bilthoven, The Netherlands; 2 Public Health Service Amsterdam, Amsterdam, The Netherlands; 3 Public Health Service Twente, Enschede, The Netherlands; 4 Public Health Service Hollands Noorden, Alkmaar, The Netherlands; 5 Public Health Service Kennemerland, Haarlem, The Netherlands; 6 Julius Centre for Health Sciences and Primary Care, University Medical Centre Utrecht, Utrecht, The Netherlands; 7 Department of Interdisciplinary Social Science, Faculty of Social and Behavioural Sciences, Utrecht University, Utrecht, The Netherlands

**Keywords:** Chlamydia trachomatis, Sexually transmitted diseases, Reinfection, Psychological determinants, Sexual behaviour, Behaviour change, STI clinic, Mathematical model

## Abstract

**Background:**

*Chlamydia trachomatis* (chlamydia), the most commonly reported sexually transmitted infection (STI) in the Netherlands, can lead to severe reproductive complications. Reasons for the sustained chlamydia prevalence in young individuals, even in countries with chlamydia screening programs, might be the asymptomatic nature of chlamydia infections, and high reinfection rates after treatment. When individuals are unaware of their infection, preventive behaviour or health-care seeking behaviour mostly depends on psychological determinants, such as risk perception. Furthermore, behaviour change after a diagnosis might be vital to reduce reinfection rates. This makes the incorporation of psychological determinants and behaviour change in mathematical models estimating the impact of interventions on chlamydia transmission especially important. Therefore, quantitative real-life data to inform these models is needed.

**Methods:**

A longitudinal cohort study will be conducted to explore the link between psychological and behavioural determinants and chlamydia (re)infection among heterosexual STI clinic visitors aged 18–24 years. Participants will be recruited at the STI clinics of the public health services of Amsterdam, Hollands Noorden, Kennemerland, and Twente. Participants are enrolled for a year, and questionnaires are administrated at four time points: baseline (before an STI consultation), three-week, six-month and at one-year follow-up. To be able to link psychological and behavioural determinants to (re)infections, participants will be tested for chlamydia at enrolment and at six-month follow-up. Data from the longitudinal cohort study will be used to develop mathematical models for curable STI incorporating these determinants to be able to better estimate the impact of interventions.

**Discussion:**

This study will provide insights into the link between psychological and behavioural determinants, including short-term and long-term changes after diagnosis, and chlamydia (re)infections. Our mathematical model, informed by data from the longitudinal cohort study, will be able to estimate the impact of interventions on chlamydia prevalence, and identify and prioritise successful interventions for the future. These interventions could be implemented at STI clinics tailored to psychological and behavioural characteristics of individuals.

**Trial registration:**

Dutch Trial Register NTR-6307. Retrospectively registered 11-nov-2016.

## Background

*Chlamydia trachomatis* (chlamydia) is the most commonly diagnosed bacterial STI among young heterosexual men and women in many western countries, including the Netherlands with up to 55,000 diagnosed infections in STI clinics nationally each year [1]. Control of this infection is of public health importance, because it can cause severe reproductive complications, including pelvic inflammatory disease (PID), ectopic pregnancy and tubal subfertility [2–5]. However, it is unclear why the prevalence of chlamydia remains unchanged even in countries with chlamydia screening programs, such as England, Australia, Canada, and the United States [6].

A difficulty in controlling chlamydia transmission is that most infections are asymptomatic [7]. Since people are unaware of their infection, initiation of preventive behaviours (i.e., condom use), or health-care seeking behaviour (i.e., chlamydia testing), mostly depends on psychological determinants, such as risk perception, self-efficacy or attitudes regarding condom use [8–11]. Previous studies have mainly focussed on identifying behavioural risk factors for chlamydia infection [12–15], while understanding how psychological determinants influences such behaviour might be more informative for the development of effective interventions [10]. For example, an increased number of sexual partners has previously been identified as a risk factor for chlamydia infection [12, 14], but having many sexual partners might not necessarily be risky if people would realistically perceive their risk for acquiring a STI and take the necessary steps to protect themselves. Therefore, studying the link between psychological determinants and behaviour and relating these to chlamydia infections might increase our understanding of chlamydia transmission. For instance, many young people tend to underestimate their personal risk of acquiring chlamydia [8, 16], which could have a negative effect on their condom use and testing uptake [8, 10, 17].

Another reason for the sustained chlamydia prevalence might be high reinfection rates after treatment or natural clearance [15, 18–20].To reduce the risk of reinfections, behaviour change (i.e., more consistent condom use) might be essential [21, 22]. Several studies have shown that individuals who were diagnosed with an STI were more likely to change into less risky sexual behaviour after they received the test results than individuals who tested negative [21, 23–27], but the influence of STI test results on underlying psychological determinants are not known. Behaviour change might be dependent on a number of psychological determinants, such as risk perception, perceived norms, perceived susceptibility, self-efficacy, knowledge, intentions, and attitudes regarding condom use [8–11, 17]. For example, while increased perceived risk of STI as a result of a positive diagnosis might induce behaviour change, receiving negative test results could lead to a false sense of security in high-risk individuals, and changing their risky sexual behaviour after the STI test may be deemed unnecessary [24]. However, regardless of the diagnosis, fear experienced before receiving the STI test results [17], might provide enough motivation to increase condom use. Quantitative longitudinal data is needed to explore the interplay between psychological and behavioural determinants after diagnosis and over time.

Longitudinal data on psychological and behavioural determinants could be used to investigate the impact of interventions aimed at reducing chlamydia (re)infections in mathematical models. Mathematical models are a tool for understanding the transmission of infectious diseases and establish a scientific basis for decision-making [28]. Predictions of the impact of interventions on prevalence arising from these models can be used to inform national health policies [29, 30]. However, psychological determinants are hardly ever incorporated in mathematical models describing STI transmission, and many models do not take into account that behaviour can change over time. Incorporating psychological determinants and behavioural change might improve the estimation of the impact of interventions on chlamydia prevalence in mathematical models. It may also increase our understanding on how to control chlamydia transmission more effectively, for example by identifying core risk groups that contribute most to transmission.

To explore the link between (changes in) psychological and behavioural determinants, and chlamydia (re)infection, a study called ‘Mathematical models incorporating Psychological determinants: control of Chlamydia Transmission’ (iMPaCT) was initiated. A longitudinal cohort study will be conducted among individuals testing for chlamydia at the STI clinic. Individual data on (re)infection rates, psychological determinants, and behaviour will be collected at different points in time to link these to chlamydia (re)infections and to study changes over time. These changes include short-term changes after a diagnosis, and long-term changes (1 year after a diagnosis at baseline). Mathematical models will be developed incorporating psychological and behavioural determinants using data from the longitudinal study. We will explore how incorporating these variables, including short- and long-term changes, influence chlamydia prevalence estimations from models.

## Methods

### Study aim

The aim of the iMPaCT study is to explore the link between psychological and behavioural determinants, and chlamydia (re)infection among heterosexuals aged 18-24 years visiting STI clinics. The following aims will be addressed:

1. To identify predictors of chlamydia infection;

- What demographic, psychological, and behavioural determinants are associated with chlamydia infection?

2. To investigate short-term and long-term changes (or stability) in psychological determinants and sexual behaviour over time;

- What is the influence of a chlamydia test result (positive or negative) on psychological determinants and subsequent sexual behaviour?
- Regarding these determinants, does change (or stability) in psychological and/or behavioural determinants affect the probability of reinfection?
- How do psychological and behavioural determinants change over time during 1 year of follow-up?

3. To explore the influence of psychological determinants on the predicted impact of intervention measures to reduce chlamydia transmission by mathematical models;
4. To explore the influence of changes in psychological determinants and sexual behaviour on the predicted impact of intervention measures to reduce chlamydia transmission by mathematical models.

### Design

A longitudinal cohort study will be conducted among young heterosexual STI clinic visitors in the Netherlands.

### Setting

Participants will be recruited from STI clinics of the Public Health Services (GGD) of Amsterdam, Kennemerland, Hollands Noorden, and Twente. In 2015, these STI clinics tested around 20,000 heterosexual men and women under the age of 25 for chlamydia according to the national registry. The majority of this group was female, ≥ 20 years old, and Dutch, and approximately 15% tested positive for chlamydia [31].

### Study population

Heterosexual men and women aged 18 to 24 years visiting the STI clinic of the GGD Amsterdam, Kennemerland, Hollands Noorden, or Twente are eligible to participate. All enrolled individuals will be invited for follow-up data collection moments, irrespective of their test result at baseline. Individuals, who are not living in the Netherlands, are not able to read or speak Dutch, commercial sex workers, men who have sex with men (including men who have sex with both men and women), and women who have sex with women, will be excluded from participation in this study. Women who have sex with both men and women will only be excluded if their last three partners were women.

### Recruitment

Participants will be recruited during the process of making an appointment at the STI clinic. To fit the study into the daily flow of the STI clinics, two different procedures will be applied. At the GGD Amsterdam, Kennemerland, and Hollands Noorden, individuals who are eligible to participate will be invited during the process of making an appointment online. Individuals will receive information about iMPaCT when they confirm their appointment. At the GGD Twente, the receptionist will invite individuals who are eligible to participate when they are making an appointment by telephone, and send them an email with information about iMPaCT. Recruitment is expected to take approximately 6 to 8 months.

### Inclusion and follow-up

**Fig. 1:**
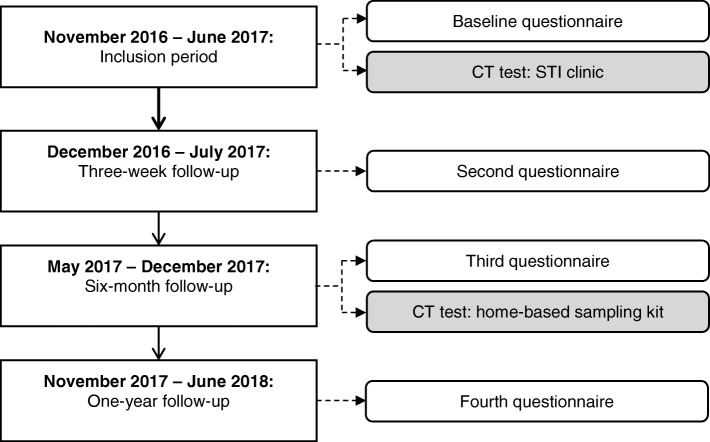
Flowchart of the longitudinal cohort study. CT = *Chlamydia trachomatis*

Participants will be enrolled for 1 year, and data on (re)infection rates, psychological determinants, and behaviour will be collected at four different points in time to link these to chlamydia (re)infections and study changes (or stability) in psychological determinants and sexual behaviour over time. Data collection will occur at the following time points: at baseline, three-week follow-up, six-month follow-up and at one-year follow-up (Fig. 1).

At baseline, individuals eligible to participate will be invited during the process of making an appointment at the STI clinic. If an individual agrees to participate, an online questionnaire (the questionnaire is described in more detail below) will be administered, which starts after participants gave informed consent. Subsequently, participants are tested for chlamydia at the STI clinic. During the consultation at the STI clinic, the participants will receive information on prevention of STI and motivational-interviewing based counselling from the nurses. Since this might have an effect on psychological and behavioural determinants [32] and possibly lead to biased answers, participants will complete the baseline questionnaire before the consultation. Therefore, participants have approximately 1 to 2 weeks to fill out the questionnaire, between making their appointment and their STI clinic visit. Individuals who agree to participate will receive an email, as a reminder, with information about iMPaCT and a web link, which will guide them directly to the online questionnaire. Participants, who completed the questionnaire after their consultation at the STI clinic, will be excluded.

The participants will receive the chlamydia test results within 2 weeks of the STI clinic visit. Approximately 1 week after the communication of the test results (three to 4 weeks after the STI clinic visit), all participants who completed the baseline questionnaire will be invited via email to fill out the second questionnaire online. Participants, who have not finished the second questionnaire, will receive two reminders by email: 1 week and 2 weeks after the invitation for the second questionnaire.

The third data collection moment will take place 6 months after baseline, because reinfections usually occur within 6 months after the initial infection [15, 18, 33, 34]. Firstly, all participants who completed the baseline questionnaire will be invited via email to fill out an online questionnaire. Additionally, all participants will be invited for a retest, irrespective of the test result at baseline. They will receive a self-swab test-kit at their home address or another preferred address, as specified in the third questionnaire, in a plain package fitting letterboxes. In this package, the participants will find simple instructions about the type of sample that they need to provide (urogenital test only, urine sample for men and vaginal swab for women) and how to collect these samples. Subsequently, the participants mail the testing-kits directly to the laboratory for chlamydia and gonorrhoea testing. The results will be communicated within three working days after the arrival of their material in the laboratory, via email with a link to a secured webpage, where the participants can download the results of their test with the login data attached in the email. Participants, who have not finished the online questionnaire, will receive two reminders by email: 1 week and 2 weeks after the invitation for the third questionnaire. Participants, who have completed the online questionnaire, but who have not mailed the test-kit to the laboratory, will receive two reminders by email: 2 weeks and 4 weeks after the test-kits are sent. The number of reminders for the retest will be the same as the number of reminders for the questionnaires, but the reminders will be spread further apart due to the possibility of delays in the logistics of the retest. Participants, who test positive, will be advised to make an appointment with their GP or at the STI clinic for appropriate treatment. A letter for the GP or STI clinic with information about the iMPaCT study, and a copy of the laboratory results will be provided.

Finally, 1 year after baseline, all participants who completed the baseline questionnaire will be invited via email to fill out the fourth online questionnaire. Participants, who have not finished the online questionnaire, will receive two reminders by email: 1 week and 2 weeks after the invitation for the fourth questionnaire. After completing the last questionnaire, irrespective of completing the three-week and six-month follow-up questionnaires, participants will receive a gift voucher with a value of €5 within a week (by mail).

### Demographic and consultation information

National STI surveillance data will be used to complement the information gathered in the questionnaires. This surveillance data includes routinely registered data of all consultations from all STI clinics in the Netherlands, such as demographic information (i.e. age, sex, ethnicity and level of education), previous testing behaviour, previous STI diagnosis, reason for testing (i.e., notified by chlamydia-positive partner, symptoms), several behavioural determinants and STI test results. In the surveillance data, each STI clinic visitor has an anonymous identification (ID) number. This ID number will automatically be incorporated in the web link to the questionnaire, which enables linkage of the surveillance data to the questionnaire. To identify chlamydia reinfections before the retest at six-month follow-up, information on STI clinic visits between baseline and six-month follow-up of the participants will also be extracted from the national STI surveillance data using this ID number, including the reason for their visit and the test result.

### Laboratory testing

The chlamydia-test performed at baseline is routine practice of the consultation at the STI clinic, using nucleic acid amplification tests (NAAT) to detect chlamydia, such as Transcription-mediated amplification (TMA) or polymerase chain reaction (PCR). Six months after baseline, all participants will be invited for a retest, irrespective of the test result at baseline. Home-based sampling and returning test kits to a laboratory by mail can be used to test for STI, such as chlamydia and gonorrhoea [18, 35–39]. The testing-kits can be mailed directly to the laboratory using the pre-paid return envelope provided. All samples will be tested for chlamydia and gonorrhoea using NAAT/PCR, with respectively a sensitivity of 97.0 and 99.3% and specificity of 98.9 and 99.3% [38].

### Defining chlamydia (re)infection

Chlamydia infection at baseline will be defined as confirmed positive NAAT-results for *Chlamydia trachomatis* at any anatomic location (urogenital, anorectal, pharyngeal) at the STI clinic. Chlamydia reinfection will be defined as confirmed positive NAAT-result for *Chlamydia trachomatis* based on either the samples provided by the participants at six-month follow-up through the self-swab testing kit (urogenital only) and/or a test at the STI clinic between baseline and six-month follow-up at any anatomic location (urogenital, anorectal, pharyngeal).

### Questionnaires

We have conducted a pilot survey in May 2016 among 296 heterosexuals aged 16–34 to evaluate the conciseness and the comprehensibility of the online questionnaire using factor and reliability analysis and comments from the respondents. People were recruited via social media and at a vocational school in Amersfoort, the Netherlands. The duration of the questionnaires in the iMPaCT study was estimated based on the results from this pilot survey; the questionnaire at baseline will take about 10–15 min and the other questionnaires will take less than 10 min. All questionnaires will be sent via Formdesk [40], a tool to create and manage online forms. Each participant has an anonymous study ID number, which will be used to link the questionnaire data from Formdesk to the information in the national STI surveillance data.

The baseline questionnaire has two parts: one part on psychological determinants, and one part on sexual behaviour. Psychological determinants included in the questionnaires that might play a role in acquiring STI are; risk perception [41–45], impulsiveness [46–50], intentions regarding condom use [11, 51], attitudes regarding prevention of chlamydia [52, 53], health goals [9, 51], knowledge [11], stigma [54–56], shame [54–56], anxiety [54–56], self-efficacy [43, 57], social support [11], peer norms [11] and self-esteem [58]. These determinants were included in the questionnaire based on associations in the literature with sexual risk behaviour, testing-behaviour, and behavioural change. Answers to these questions are measured on a 5-point Likert scale.

The questions about sexual behaviour are based on several validated questionnaires, including questionnaires from previous STI studies [11, 59–61], and the UK NATSAL [13]. Participants will be asked about the number of sex partners, age at sexual debut, condom use, and we will pose detailed questions on the last three partners, including begin and end of the partnership, condom use, sex frequency, type of sex, and partner characteristics.

The follow-up questionnaires will be the same as the baseline questionnaire, with a few additional questions. To explore short-term effects of diagnoses (and if applicable treatment) on possible changes in psychological and sexual behaviour determinants, the questionnaire at three-week follow-up will include the same questions on psychological determinants and sexual behaviour as the baseline questionnaire. For participants who test positive at baseline, questions are added about partner notification in the week following treatment. In the questionnaire at six-month and one-year follow-up, questions will be added about visits to the STI clinic between baseline and six-month follow-up and between six-month follow-up and one-year follow-up, including test results, treatment, and reasons for the visit(s).

### Expected response

Based on the number of consultations at the STI clinics of the public health services in Twente, Hollands Noorden, Kennemerland and Amsterdam in the last 6 months of 2015 in the national registry, around 10,000 heterosexual STI clinic visitors will meet the inclusion criteria during 6 months of recruitment (40% men and 60% women). Table 1 shows the expected response for each data collection moment by STI clinic. The response rate at baseline is expected to be low, because recruitment at baseline is internet-mediated (passive recruitment) and there will be no face-to-face interaction with potential participants [62, 63]. We aim to include 2000 people at baseline (20% response rate), of which 800 are men and 1200 are women, based on the distribution of heterosexual male and female visitors at the STI clinics [31]. We expect 15% of the participants at baseline to test positive (*N* = 300) [31].

**Table 1:** Expected number of participants per STI clinic at each follow-up moment

STI clinic	Expected number of participants					
	Baseline (men/women)	Baseline Ct+	Three-week	Six-month	Six-month Ct+	One-year
Twente	400 (160/240)	60	320	193	31	120
Hollands Noorden	400 (160/240)	60	320	193	31	120
Kennemerland	400 (160/240)	60	320	193	31	120
Amsterdam	800 (320/480)	120	640	385	62	240
Total	2000 (800/1200)	300	1600	964	155	600

*Abbreviations*: *Ct+* chlamydia positive

The response rates at the follow-up moments are expected to be higher than at baseline, because the participants will receive personal invitations by email instead of the impersonal baseline invitation addressed to all the STI clinic visitors who were eligible to participate. However, the response rates might decline over time [30]. At three-week follow-up, a response rate of 80% (*N* = 1600) is expected (this includes sending reminders) [30]. All participants will be contacted by email again at the six-month follow-up, also participants who did not respond to the three-week follow-up. The participation rate at six-month follow-up among people who tested chlamydia positive at baseline is expected to be around 66% [18], and the participation rate is likely to be lower for individuals who tested chlamydia negative at baseline [24] and is expected to be around 45%. Therefore, approximately 1000 participants will be tested for chlamydia at six-month follow-up. We expect 15% of the participants that were chlamydia negative (Ct-) at baseline and 20% of the participants that were chlamydia positive (Ct+) at baseline to test positive at six-month follow-up (*N* = 155) [31, 33, 34]. All participants who completed the baseline questionnaire will be contacted again at the one-year follow-up, also participants who did not respond to the three-week follow-up and/or the six-month follow-up, and we expect a response rate of 30% (*N* = 600). Participants can choose to leave the study at any time for any reason. If participants formally withdraw from the study by email, they will not be invited for follow-up data collection.

### Sample size and power calculations

To explore if the study population will be large enough to detect differences in psychological and behavioural determinants between baseline and six-month follow-up with adequate statistical power, sample size and statistical power calculations were performed in Stata version 13.0 [64]. Firstly, sample size calculations for at least 80% power were performed for the participants who tested positive (Ct+) and participants who tested negative for chlamydia at baseline (Ct-), assuming a type I error (α) of 0.05, 34% loss to follow-up after 6 months in the Ct + group, and 55% loss to follow-up after 6 months in the Ct- group (based on the expected response rates described above). Secondly, power calculations were performed with the expected sample size of 2000 participants at baseline, assuming 15% chlamydia positivity (*n* = 300) [31].

The sample size and power calculations for sexual behaviour were calculated with condom use as an example. Soetens et al., (2015) [26] found that condom use 1 year after the chlamydia test at baseline increased in the Ct + group and decreased in the Ct- group. Based on these results, the percentage of participants in our study using a condom with the last sexual contact was assumed to increase in individuals after a Ct + baseline test and decrease in individuals after a Ct- baseline test. The sample size and power for sexual behaviour were calculated for three different scenarios: 10 (scenario 1), 15 (scenario 2), or 20 (scenario 3) percent change at six-months follow-up (Table 2).

**Table 2:** Sample size and power calculations for different scenarios

	Change (%)	Sample size needed at baseline (80% power)		Expected power with sample size *n* = 2000 at baseline	
		Ct+	Ct-	Ct + (n = 300)	Ct- (*n* = 1700)
Scenario 1	10%	470	611	60%	> 99%
Scenario 2	15%	220	286	91%	> 99%
Scenario 3	20%	129	167	> 99%	> 99%

Sample size and power calculations to detect a change in psychological determinants or sexual behaviour at six-month follow-up with at least 80% power, assuming 34% loss to follow-up in the chlamydia positives (Ct+) and 55% loss to follow-up in the chlamydia negatives (Ct-)

*Abbreviations*: *Ct+* chlamydia positive, *Ct-* chlamydia negative

This is, to our knowledge, the first study assessing changes in psychological determinants after diagnosis of chlamydia or over a longer period of time. Therefore, no literature is available to inform possible changes in psychological determinants in our sample size calculations. The sample size and power calculations for the psychological determinants were calculated with risk perception as an example, but this can also be generalised to other psychological determinants. Research has shown that people chronically underestimate their personal risk of acquiring chlamydia [8]. Tailored risk information (i.e. after a consultation at the STI clinic) might increase an individual’s perceived risk for STI [65]. Furthermore, individuals with previous STI diagnoses are more likely to report higher perceived risk for STI than those with no previous STI diagnoses [66, 67]. Therefore, we hypothesized that risk perception, which will be assessed in the questionnaire as the estimated personal risk of chlamydia on a scale from 0 to 100, might increase in individuals after a Ct + baseline test and decrease in individuals after a Ct- baseline test. The sample size and power for the psychological determinants were calculated with the same hypothesized percent change as for sexual behaviour (Table 2).

The expected sample size of 2000 participants will be large enough to detect a change of 10–20% in psychological determinants and behavioural with adequate statistical power (power > 80%). The expected sample size of 300 in the Ct + group is too small for detecting 10% change (scenario 1) for ≥70% power, but the expected sample size is sufficient for detecting ≥15% change (scenario 2 and 3), which might be more likely in this group [26].

### Statistical analyses

The main analysis will include all participants who completed the baseline questionnaire, irrespective of their test result. Possible response bias will be explored in a (non-)response study using anonymised national STI surveillance data of all individuals eligible to participate who visited the STI clinic in the inclusion period. Demographic characteristics, sexual behaviour, and STI consultation information will be compared between participants who completed the baseline questionnaire, and all the STI clinic visitors who were invited to participate, but did not complete or start the baseline questionnaire. The participants who completed the baseline questionnaire will be identified in the surveillance data, using the previously described ID number incorporated in the web link to the questionnaire.

We expect only few missing values in the completed baseline questionnaires, because each question has to be answered before the next question appears. Furthermore, data consistency checks will be incorporated in the online questionnaire. Missing values in variables extracted from the national STI surveillance data or in the second, third and last questionnaire due to loss to follow-up will be included as a separate category if more than 5% is missing.

Baseline characteristics of the study population will be presented, using summary statistics, including means, standard deviations, medians and ranges for continuous variables and frequency distributions for binary and categorical variables. To identify predictors of participation in the (non-)response study, and to identify predictors of chlamydia (re)infection, univariable and multivariable logistic regression analysis will be performed. In the univariable analysis, variables significantly associated with the outcome (participation or chlamydia (re)infection) will be included in the multivariable models. Multivariable models will be constructed using a backward elimination procedure. Statistical significance will be defined as a *p*-value ≤0.1, and odds ratios and 95% confidence intervals of each predictor variable will be reported. Covariates based on a priori hypotheses will be examined as potential confounder or effect modifiers in the models.

To identify distinct risk groups for chlamydia (re)infection, based on the results of the multivariable logistic regression analysis, we will use latent class analysis for multivariable categorical data. In this analysis, underlying dimensions (latent classes) of the dependent variables can be inferred based on patterns in the observed data. The latent classes arising from this analysis could be combinations of several measured psychological, behavioural and demographic variables. Covariates that are independent of the outcome, but might influence the latent classes will be included in the analysis. The number of latent classes will be determined by increasing the number of classes until the best fitting model has been found, using the Bayesian Information Criterion (BIC) to assess the goodness of fit. The latent classes can be used to define distinct sexual risk profiles, which can be implemented in future mathematical models.

To explore changes in psychological determinants and sexual behaviour over time using data from the three-week, six-month, and one-year follow-up moments, and to identify risk profiles for chlamydia reinfection, we will use latent transition analysis, which is an extension of the latent class analysis described above. In this analysis, movement from one latent class to another over time can be determined. Similar to the latent class model, the BIC will be used to assess how well the latent transition model fits the observed data. The estimated transition probabilities can be implemented in future mathematical models of the transmission of chlamydia, which might enable us to better capture the complexity of sexual behaviour.

### Mathematical model

The mathematical model will be a pair compartmental model representing a heterosexual population of men and women aged 18–24 years. Chlamydia will be described with a susceptible-infected-susceptible (SIS) structure. The infection parameters for chlamydia are reasonably well established and will be used from the available literature. The transmission rate per sex act will be calibrated to the positivity rate found at baseline. The model population will be subdivided into risk groups according to the risk classes identified in the latent class model (based on psychological and behavioural determinants from the baseline questionnaire).

Behavioural change after a diagnosis and in time will be incorporated by moving people from one risk group to another based on the latent transition model. First, we will explore the influence of a diagnosis on short-term changes of psychological and behavioural determinants in the model. This data will be based on differences between the baseline, three-week, and the six-month follow-up questionnaires. Second, we will theoretically explore the effect of long-term behaviour change on the impact of intervention measures, using the questionnaire data at one-year follow-up.

## Discussion

The iMPaCT study will provide insights into the link between psychological determinants and sexual behaviour, behavioural change, and chlamydia (re)infections. We propose that incorporating these determinants in mathematical models will improve the impact assessment of interventions aimed at reducing chlamydia transmission. Chlamydia interventions that have been applied in practice have mainly focused on increasing testing uptake, and previous mathematical modelling studies have shown that, depending on the coverage of chlamydia testing in the general population, testing and treatment could be an effective strategy to reduce chlamydia prevalence, [30, 68, 69]. However, empirical studies have established that the coverage of chlamydia testing has not been high enough to observe a significant reduction in the population prevalence of chlamydia [13, 61, 70]. Therefore, a paradigm shift is needed to control chlamydia transmission more effectively. For example, interventions could be focused on increasing testing uptake among core risk groups based on psychological and behavioural characteristics to prevent reinfection after a diagnosis. Our mathematical model informed by the data of the cohort study will be able to estimate the impact of such interventions on chlamydia prevalence, and identify and prioritise successful interventions for specific risk groups, which might lead to more efficient ways to control chlamydia transmission. Subsequently, these interventions could be implemented at STI clinics tailored to psychological and behavioural characteristics of individuals.

### Strengths

This is the first longitudinal cohort study investigating short-term and long-term changes in psychological determinants and sexual behaviour after chlamydia diagnosis. The prospective study design of this study with repeated measurements, namely the follow-up questionnaires and the retest, and the combination of an extensive selection of psychological determinants will expand our knowledge of risk factors for (re)infection. Another strength of this study is the combination of two data sources: longitudinal questionnaire data on psychological determinants and sexual behaviour, and national STI surveillance data on demographics, previous testing behaviour, and laboratory confirmed STI diagnosis. Therefore, we are able to obtain extensive knowledge with a relatively concise questionnaire, because questions on demographics, previous testing behaviour, STI diagnosis and other variables that are available in the national STI surveillance could be omitted in the online questionnaire.

The pilot survey, which has been conducted a few months before the start of iMPaCT study, enabled us to improve the comprehensibility of the questionnaire. This, in combination with sending reminder emails and offering incentives (gift voucher, free home-based sampling kit), might lead to higher response and completion rates [71]. Furthermore, the psychometric evaluation of the pilot survey and the advantages of using online questionnaires, such as programmed warning alerts to prevent incorrect answers (i.e., number of partners last 6 months can’t be higher than the number of partners in the last year), ensured optimal reliability and validity of the longitudinal questionnaire data.

Finally, most mathematical models for infectious disease transmission incorporating behaviour change are entirely theoretical and lack validation with empirical data [72]. Our mathematical model will be informed by real-life data on behaviour change, which might result in more realistic model estimations and the opportunity to validate the model outcomes. The model outcomes could be directly translated into to advice for public health policy makers about effective intervention measures.

### Limitations

First, the questionnaire data is self-reported, which could lead to reporting bias, such as under- or over-reporting of sexual behaviour. Although sexual behaviour in the national STI surveillance data is also self-reported, and thus prone to bias, sexual behaviour in the surveillance data will be matched to sexual behaviour as reported in the questionnaire to check for consistency. Furthermore, being notified for an STI or having STI-related symptoms might affect answers in the questionnaire [54, 67] and this will be taken into account in the statistical analyses. Response bias may also occur, and we will assess this in a (non-)response study by extracting the iMPaCT participants from the national STI surveillance data, and compare demographics, sexual behaviour, and STI consultation information between the participants and all eligible STI clinic visitors who were invited to participate, but did not complete or start the baseline questionnaire. We will use this (non-)response study to estimate the generalizability of the iMPaCT study population with reference to all young heterosexual STI clinic visitors and to guide the interpretation of the results. The iMPaCT study population is not likely to be representative of the general population, as STI clinic visitors tend to be more high-risk compared to the general population. However, this group potentially benefits the most from improved interventions. Thus, in this study we will gather detailed information for exactly the group of interest.

Previous longitudinal chlamydia studies have shown that response rates decline over time and our study will most likely not be an exception [18, 30]. To minimize loss to follow-up, free home-based sampling kits and promised monetary incentives will be used to encourage participation rates at six-month and one-year follow-up. The samples size and power calculations, taking loss to follow-up into account, showed that through recruitment at multiple STI clinics in different regions of the Netherlands, a sufficiently large and nationally representative group of STI clinic visitors can be approached for participation in the iMPaCT study.

It is likely that periods of high and low risk behaviour alternate during individual sexual careers [73], such as a period of high risk sexual behaviour after separating from a long standing partnership. Therefore, the timing of the follow-up data collection moments is crucial. For example, it could be argued that the period between the follow-up questionnaire after 3 weeks and baseline is too short to detect changes in sexual behaviour, and the questionnaires 6 months and 1 year after baseline might not be long-term enough to capture changes in people’s behaviour that are not necessarily offset by an event such as a diagnosis. However, we speculate that the effect of a positive STI diagnosis on psychological determinants, such as intentions and attitudes regarding condom use, might be strongest in the first few days after receiving the test results. Furthermore, the optimal timing of testing for reinfections is not known, and the recommended timing of retesting across different countries ranges between 3 to 12 months [74, 75]. Therefore, participants will be invited for the retest 6 months after the baseline chlamydia test combined with a questionnaire, and after the same length of time (6 months after the retest), the participants will be invited to fill out the last questionnaire at one-year follow up.

## Abbreviations

BIC: Bayesian information criterion
Ct: *Chlamydia trachomatis*
GGD: Public health services (in Dutch: Gemeentelijke Gezondheids Dienst)
ID: Identification number
NAAT: Nucleic acid amplification test
NATSAL: The national survey of sexual attitudes and lifestyles
PCR: Polymerase chain reaction
PID: Pelvic inflammatory disease
STI: Sexually transmitted infection
TMA: Transcription-mediated amplification
